# Infliximab, a Monoclonal Antibody against TNF-α, Inhibits NF-κB Activation, Autotaxin Expression and Breast Cancer Metastasis to Lungs

**DOI:** 10.3390/cancers16010052

**Published:** 2023-12-21

**Authors:** Anjali Shinde, Xiaoyun Tang, Rajesh Singh, David N. Brindley

**Affiliations:** 1 Department of Biochemistry, Faculty of Science, The MS University of Baroda, Vadodara 390002, Gujarat, India; anjalishinde4891@gmail.com; 2 Cancer Research Institute of Northern Alberta, Department of Biochemistry, University of Alberta, Edmonton, AB T6G 2S2, Canada; xtang2@ualberta.ca; 3 Department of Molecular and Human Genetics, Banaras Hindu University (BHU), Varanasi 221005, Uttar Pradesh, India

**Keywords:** anti-TNF-α therapy, breast cancer, inflammation, lysophosphatidic acid, NF-κB, tumor growth

## Abstract

**Simple Summary:**

Breast cancer is a heterogeneous pathological condition involving multiple cytokines and inflammation in the tumor microenvironment (TME). Blocking TNF-α signaling with Infliximab, a monoclonal antibody, decreased breast cancer metastasis to lungs in mice by ~60%. Decreasing TNF-α signaling in this aggressive 4T1 breast cancer model decreased NF-κB activation and the expression of inflammation-related genes that regulate metastasis. Attenuating TNF-α signaling decreased the activation of the autotaxin–lysophosphatidate–inflammatory cycle, which provides a strategy for decreasing breast cancer metastasis.

**Abstract:**

An inflammatory milieu in the tumor microenvironment leads to immune evasion, resistance to cell death, metastasis and poor prognosis in breast cancer patients. TNF-α is a proinflammatory cytokine that regulates multiple aspects of tumor biology from initiation to progression. TNF-α-induced NF-κB activation initiates inflammatory pathways, which determine cell survival, death and tumor progression. One candidate pathway involves the increased secretion of autotaxin, which produces lysophosphatidate that signals through six G-protein-coupled receptors. Significantly, autotaxin is one of the 40–50 most upregulated genes in metastatic tumors. In this study, we investigated the effects of TNF-α by blocking its action with a monoclonal antibody, Infliximab, and studied the effects on autotaxin secretion and tumor progression. Infliximab had little effect on tumor growth, but it decreased lung metastasis by 60% in a syngeneic BALB/c mouse model using 4T1 breast cancer cells. Infliximab-treated mice also showed a decrease in proliferation and metastatic markers like Ki-67 and vimentin in tumors. This was accompanied by decreases in NF-κB activation, autotaxin expression and the concentrations of plasma and tumor cytokines/chemokines which are involved in metastasis. We also demonstrated a positive correlation of TNF-α -NF-κB and ATX expression in breast cancer patients using cancer databases. Studies in vitro showed that TNF-α-induced NF-κB activation increases autotaxin expression and the clone forming ability of 4T1 breast cancer cells. This report highlights the potential role of Infliximab as an additional approach to attenuate signaling through the autotaxin–lysophosphatidate–inflammatory cycle and decrease mortality from metastatic cancer.

## 1. Introduction

Inflammation is intrinsically linked to breast cancer progression and metastasis [1,2,3,4,5]. It is one of the major hallmarks of cancer which modulates the tumor microenvironment (TME) [6,7,8,9,10]. The major factors responsible for inflammation are invading immune cells and various cytokines and chemokines released into the TME. TNF-α, a proinflammatory cytokine, is associated with disease progression, and it plays a vital role in regulating various inflammatory pathways involving NF-κB [11,12,13,14]. Cancer and stromal cells in the microenvironment of breast tumors also show increased levels of TNF-α, which induces NF-κB activation and enhances the proliferation of breast cancer cells, cell survival and metastasis [15,16,17,18]. NF-κB-responsive genes are associated with both cell survival and cell death in inflammatory conditions. Dysregulation of the NF-κB pathway in the TME can shift the equilibrium toward tumor progression and cancer metastasis [19,20,21].

Autotaxin (ATX) is a secreted enzyme that is a key regulator of lysophosphatidate (LPA) production from lysophosphatidylcholine. LPA is degraded by the actions of three lipid phosphate phosphatases, and it signals through six G-protein-coupled receptors, which stimulates the division, survival and migration of breast cancer cells [22,23]. *ATX* is among the 40–50 most upregulated genes in metastatic tumors; it enhances inflammation and stabilizes blood vessels in the core regions of tumors and for the angiogenesis that contributes to tumor growth [24]. Hence, it is important to investigate the molecular events that produce ATX, which is responsible for excess LPA production, enhancing tumor growth, metastasis and resistance to chemotherapy and radiotherapy [25,26]. ATX and LPA enhance the activation of NF-κB and the increased production of multiple inflammatory mediators, including TNF-α [27,28]. These mediators increase further ATX transcription and secretion in a feedforward inflammatory cycle in several tumors including breast cancer and hepatocellular carcinoma [23,29]. Thus, in terms of cancer treatments, additional therapeutic approaches could involve interventions that attenuate signaling through the ATX-LPA-inflammatory cycle [25,30]. This should be possible by decreasing inflammation and interrupting the cycle as an approach to lowering ATX production.

We investigated this role of inflammation in ATX and LPA signaling in the present study and established a link among TNF-α, NF-κB and ATX in breast cancer. Infliximab is a monoclonal antibody against TNF-α which was used to neutralize activity by binding to soluble and transmembrane forms of TNF-α and stopping interactions with its receptors in a syngeneic model of breast cancer in BALB/c mice to understand its primary effect on tumor progression. Infliximab had a significant effect inhibiting breast tumor growth, but more importantly, it decreased metastasis to lungs by ~60%. Infliximab also decreased NF-κB activation and the expression of NF-κB-responsive genes. Furthermore, we demonstrated that Infliximab decreased ATX activity in the primary tumor and the expression of genes that regulate the epithelial–mesenchymal transition (EMT). A large-scale analysis of human breast cancer using the TIMER and GEPIA2 databases showed a positive correlation among TNF-α, RELA, or v-rel avian reticuloendotheliosis viral oncogene homolog A and ATX in breast cancer patients. Overall, this study adds to our understanding of the molecular mechanism underlining TNF-α-induced NF-κB activation and links it to increased ATX-LPA-inflammatory signaling and metastasis.

## 2. Materials and Methods

### 2.1. Reagents

This study used Anti-p-IκBα (Cell Signaling Technology, Danvers, MA, USA), Anti-Actin (Cell Signaling Technology, Danvers, MA, USA), Anti-p65 (Cell signaling Technology, Danvers, MA, USA), Anti-Vimentin (Cell Signaling Technology, Danvers, MA, USA) and Ki-67 (Cell Signaling Technology, Danvers, MA, USA) and secondary anti-rabbit and anti-mouse antibodies (Invitrogen, Carlsbad, CA, USA). Anti-ATX was gifted by Dr. T. Clair (National Cancer Institute, Bethesda, MA, USA), and IOA-289 was a gift from iOnctura (Genève, Switzerland).

### 2.2. Animals

All procedures were performed in accordance with the guidelines of the Canadian Council of Animal Care and were approved by the University of Alberta Animal Welfare Committee. Female BALB/c mice were obtained from Charles River (Kingston, ON, Canada), and they were used at 10 weeks of age. The mice were kept in conditions of 21 °C, 55% humidity and a 12 h light–dark cycle. The mice were fed with a 4% fat laboratory diet and water ad libitum.

### 2.3. Orthotopic Breast Cancer Models in Syngeneic Mice

Mouse 4T1 breast cancer cells were cultured in high-glucose DMEM (Sigma Life Science, St. Louis, MO, USA) and kept at 37 °C, 5% CO_2_ and 95% humidity. First, 2 × 10^5^ cells/mL were suspended with 50% Matrigel (BD Biosciences, Mississauga, ON, Canada) in PBS, and 20,000 cells were injected using a 30-gauge needle in the 4th left inguinal mammary fat pad of female BALB/c mice [31]. Infliximab was administered i.p. at a recommended dose of 10 mg/kg and thereafter at intervals within 7 days of each other in 100 µL of physiological saline [32] These injections occurred on day 7 after tumor cell inoculation when the tumors became palpable and then on days 13 and 21. Control mice were injected with the same volume of saline on the same days. Each group consisted of 9 mice. Tumor volumes were measured using calipers from 4–5 days following the injection of cancer cells, and this continued until the mice were euthanized on day 24.

E0771 breast cancer cells (1.0 × 10^6^) were inoculated as above into the 4th left inguinal mammary fat pad of female C57BL/6J-ENPP2fl/flAdipoq-Cre+ (ATX-KO) and C57BL/6J-ENPP2fl/fl (control) mice [30]. The mice were maintained as described above. IOA-289 was administered orally at 100 mg/kg twice per day as described earlier [30]. Control mice were treated orally at the same times with the vehicle.

### 2.4. Lung Nodule Measurement

Formalin-perfused lungs were stained with India ink. Visible metastatic nodules were counted on each lobe.

### 2.5. Immunohistochemistry

IHC was performed on 5 µm paraffin-embedded tumor sections using a Dako LSAB+ Universal Kit. Antigen retrieval was performed by microwaving hydrated slides in 10 mM sodium citrate buffer (10 mM sodium citrate, 0.05% Tween 20; pH 6.0) for 20 min. Primary antibodies against ATX, p65, Ki-67 and Vimentin were used for IHC staining, following the manual of the kit. Then, 10–15 images per section were taken using a Zeiss Axioskop 2 imaging system. The staining intensities of the proteins of interest were quantified as the integrated density using ImageJ software (version: 1.8.0_172).

### 2.6. Western Blotting

Tissue lysates were prepared in a RIPA lysis buffer (150 mM NaCl, 50 mM Tris-Cl, 1% NP40, 0.5% sodium deoxycholate, 10% SDS, and protease inhibitor cocktail). Protein concentrations were determined using a Pierce BCA protein estimation assay kit (Thermofisher, Pleasanton, CA, USA) and equal amounts of protein were loaded on a 10% SDS-PAGE gel. Protein was electro-blotted on PVDF membranes at 100 V for 1 h at 4 °C. Following the transfer, the membrane was treated with blocking buffer (Odyssey Blocking buffer (PBS), Thermo Fisher Scientific, London, UK) for 1 h at room temperature. The membrane was incubated overnight with specific primary antibodies. After incubation, the membrane was washed three times for 10 min with PBS-T (PBS containing 0.1% Tween 20) and incubated with a secondary antibody (Invitrogen, USA) at room temperature for 1 h. The membrane was again washed three times with PBS-T, and protein bands on the membrane were then visualized using an LI-COR Odyssey Imaging System.

### 2.7. RT-PCR

Total RNA was isolated using Biobasic Molecular Biology kit, followed by cDNA synthesis using a BlasTaq 2× qPCR MasterMix (Applied Biological Materials Inc., Richmond, BC, Canada). Primers for genes of interest were designed using the IDT primer designing tool (https://www.idtdna.com/scitools/Applications/RealTimePCR/default.aspx, accessed on 23 November 2022) for PCR. The levels of mRNAs were determined via the 2^−ΔΔCT^ method, using *cyclophilin A*, *glyceraldehyde phosphate dehydrogenase* (*GAPDH*) *or β-actin* as endogenous controls. Primer sequences are verified using Blast, and they are listed in Supplementary Table S1. The melt curves were also acquired. We had established previously that similar RT-PCR results were obtained when *GAPDH* was used as a housekeeping gene instead of *cyclophilin A* [23]. We are aware that *cyclophilin A* expression is reported to increase in certain cancers [33,34,35]. Therefore, we were careful to check that the conclusions from the RT-PCR using *cyclophilin A* as a housekeeping gene were replicated when we used *GAPDH* or *β-actin* as controls for the expression of IL-6, which was used as an example (Supplementary Figure S1). In addition, we verified that the results from the RT-PCR were compatible with the conclusions obtained from IHC, Western blots and activity measurements; the more meaningful expression of the proteins as described in the Section 3.

### 2.8. ATX Assay

ATX activity was measured in the primary tumor using choline release from lysophosphatidylcholine. Primary tumors were homogenized in 500 µL of buffer A (100 mM Tris-HCl, pH 9.0; 500 mM NaCl; 5 mM MgCl_2_; 0.05% *v*/*v* Triton X-100) using a Qiagen TissueLyser II system (Qiagen, Toronto, ON, Canada). Activity measurements were normalized to protein content via a BCA protein assay (Thermo Fisher Scientific, Rockford, IL, USA). In total, 5 µL of tissue lysate with an equivalent protein content was mixed with 19 µL of buffer A. Samples were incubated at 37 °C for 30 min and then mixed with 25 µL of 6 mM lysophosphatidylcholine in buffer A and incubated for 48 h for the primary tumor lysate at 37 °C. Basal choline levels were determined by adding 1 μL of a 10 mM ATX inhibitor, IOA-289, into the samples. After incubation, 20 µL samples were mixed with 90 µL of buffer C (88.3 µL of Buffer B (100 mM Tris-HCl, pH 8.5, and 5 mM CaCl_2_), 0.58 μL of 10 mM Amplex Red, 0.12 μL of 1000 U/mL horseradish peroxidase, and 1 μL of 50 U/mL choline oxidase) and incubated in the dark at room temperature for 1 h. Choline formation was measured at fluorescence, Ex544/Em590 nm, and quantified against a choline standard curve.

### 2.9. Cytokine/Chemokine Measurements

Thirty-two cytokines, chemokines and growth factors were measured in plasma and primary tumor tissue by Eve Technologies Corp. (Calgary, AB, Canada), using a Milliplex Mouse Cytokine/Chemokine 32-plex kit (Millipore, St. Louis, MO, USA), according to the manufacturer’s protocol, on a Luminex 100 system (Luminex, Austin, TX, USA). Tissue specimens (25–30 mg) were homogenized using TissueLyser II (Qiagen, Venlo, The Netherlands) in 200 μL of 20 mM Tris-HCl (pH 7.5) buffer with 0.5% Tween 20, 150 mM NaCl and protease inhibitor cocktail (HB9081, Hello Bio, Princeton, NJ, USA) and centrifuged for 10 min at 4 °C. The supernatant was transferred to a fresh tube, and the protein content was measured using the Pierce BCA protein assay kit (Thermo Fisher Scientific, Rockford, IL, USA). Samples were normalized to equal protein concentrations before analyses. Plasma samples were diluted with the same volume of PBS before measurements.

### 2.10. Clonogenic Assay

4T1 cells were seeded at a density of 2000 cells/well in 6 well plates for a colony forming assay. The cells were seeded in high-glucose DMEM with 10% FBS and treated as indicated post seeding. The colony was monitored for up to 8 days, and media were changed every 2 days along with treatments until termination. The cells were fixed with cold methanol and stained using crystal violet for visualizing the colonies, which were counted, and the size was measured using an ImageJ software (version: 1.8.0_172).

### 2.11. Database

The TIMER database (http://timer.cistrome.org/ (accessed on 23 November 2022)) is a web resource used for the systemic analysis and evaluation of clinical impacts of different immune factors in diverse cancer types [36]. Hence, we analyzed the transcript levels of genes of interest using the correlation tool in breast cancer patients (TCGA-BRCA data through the GDC data portal). The bc-GenExMiner database (http://bcgenex.ico.unicancer.fr/BC-GEM/GEM-Accueil.php (accessed on 16 October 2023).) was used to investigate the expression of genes in different subtypes of breast cancer patients. The datasets that were analyzed are listed in the Supplementary Table S2 [37]. The GEPIA2 database (http://gepia2.cancer-pku.cn/ (accessed on 23 November 2022)) was used for the correlation of genes of interest in breast tumors (TCGA and GTEX datasets) [38]. Spearman’s rho value was used as the degree of their correlation, with values lying between −1 and +1.

### 2.12. Statistics

Results are shown as mean ± SEM values for number of independent experiments. Results were analyzed using Student’s *t*-test or an ANOVA using GraphPad Prism 6 (La Jolla, CA, USA), and *p* < 0.05 was considered statistically significant. For correlation studies, a regression analysis was used to examine the relation between genes of interest as described above. Significance is indicated by * *p* < 0.05, ** *p* < 0.01, *** *p* < 0.001, and **** *p* < 0.0001.

## 3. Results

### 3.1. Effect of Infliximab on Breast Cancer Metastasis to Lungs

**Figure 1:**
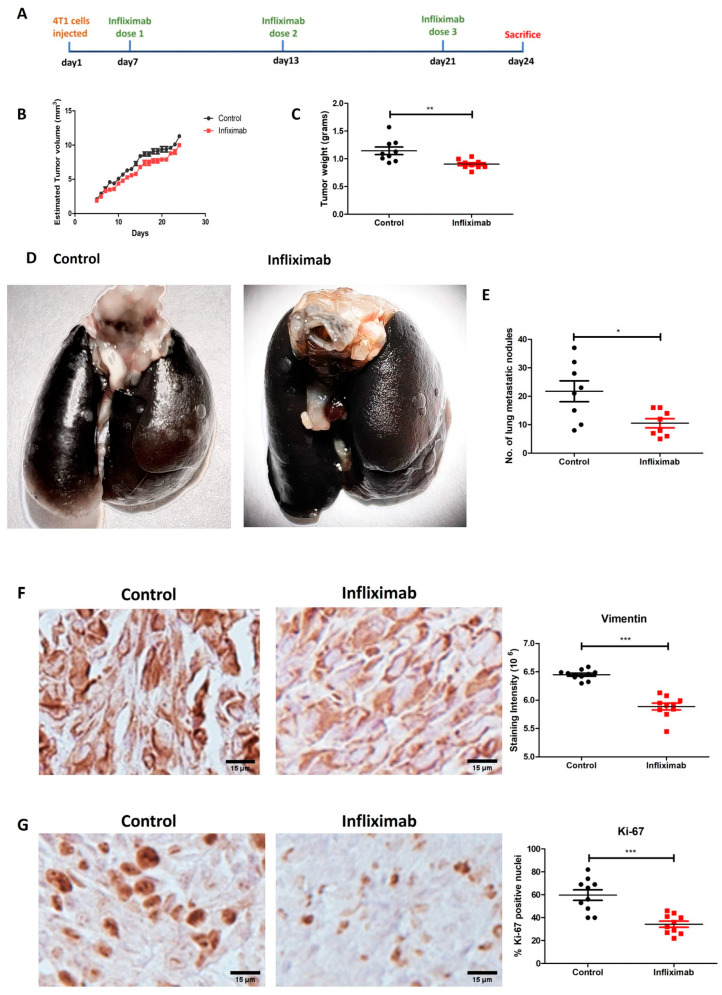
Infliximab decreases breast cancer metastasis to lungs and decreases vimentin and Ki-67. (**A**) Schematic representation of study protocol. (**B**) Tumor volume measured using calipers in control and Infliximab groups of mice. (**C**) Tumor mass excised from the control and Infliximab groups of mice on day 24. (**D**) Metastatic lung nodules in lung injected with India Ink. (**E**) Number of lung metastatic nodules in control versus Infliximab-treated mice and (**F**,**G**) expression of Vimentin and Ki-67 via IHC staining of tumors from control and Infliximab-treated mice. Significance is indicated by * *p* < 0.05, ** *p* < 0.01, and *** *p* < 0.001.

TNF-α is a major inflammatory cytokine in the tumor microenvironment, and its levels are increased in metastatic breast cancers to adversely affect disease progression. We focused on targeting TNF-α signaling in a syngeneic BALB/c mouse model using 4T1 breast cancer cells with Infliximab, a monoclonal antibody against TNF-α. Infliximab was injected into BALB/c mice after tumors became palpable on day 7 after the inoculation of 4T1 breast cancer cells into a mammary fat pad, and this was repeated on days 13 and 21 (Figure 1A). Tumor volumes (Figure 1B) appeared to be decreased marginally in the Infliximab-treated mice compared to the controls. The mass of the excised tumors, which is a more accurate measurement, was significantly decreased in the Infliximab group of mice compared to controls (Figure 1C). Our major finding was a remarkable decrease of ~60% in the number of lung metastatic nodules in the Infliximab group of mice on day 24 of the experiment (Figure 1D,E). We also observed a significant decrease in metastatic and proliferation markers in the tumors of the Infliximab group of mice compared to the ontrol mice, determined via IHC using antibodies against Ki-67 (cell proliferation) and Vimentin (epithelial-mesenchymal transition (EMT) (Figure 1F,G). Thus, Infliximab can decrease breast tumor growth, but the more important finding was the decrease in lung metastasis.

### 3.2. Infliximab Alters Concentrations of Cytokines in Plasma and Breast Tumors in a Syngeneic Mouse Model

**Figure 2:**
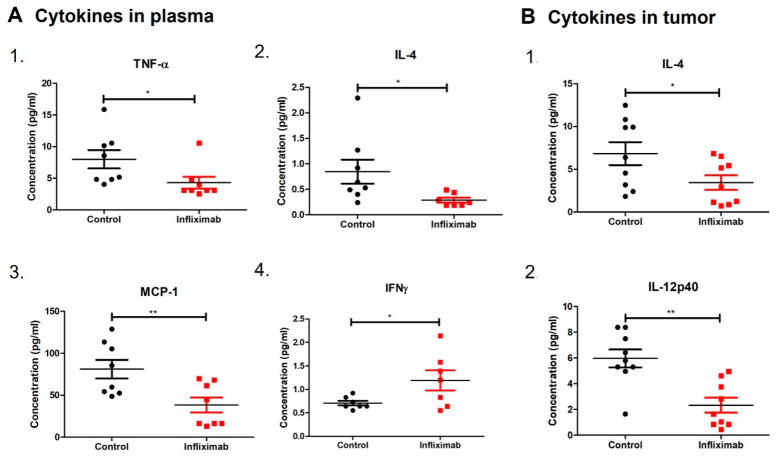
Cytokine profiles in BALB/c mice treated with Infliximab. (**A**) Concentrations of TNF-α, IL-4, MCP-1 and IFNγ in the plasma of mice treated with Infliximab and controls. (**B**) Concentrations of IL-4 and IL-12p40 in primary tumors of BALB/c mice treated with Infliximab control mice. Significance is indicated by * *p* < 0.05 and ** *p* < 0.01.

The TNF-α-activated NF-κB pathway is a one of the major inducers of inflammatory cytokines in breast cancer. Therefore, we evaluated the concentration of cytokines/chemokines/growth factors in plasma and the primary tumors of Infliximab-treated mice compared to control mice. Infliximab treatment decreased the levels of TNF-α, IL-4 and MCP-1 in plasma compared to controls (Figure 2A). We also observed a significant increase in the plasma levels of IFNγ in the Infliximab group of mice compared to the controls. Primary tumors showed significant decreases in IL-4 and IL-12p40 in Infliximab-treated mice (Figure 2B).

### 3.3. Infliximab Treatment Decreases Inflammation-Related Genes and NF-κB Activation in Breast Tumors in a Syngeneic Mouse Model

**Figure 3:**
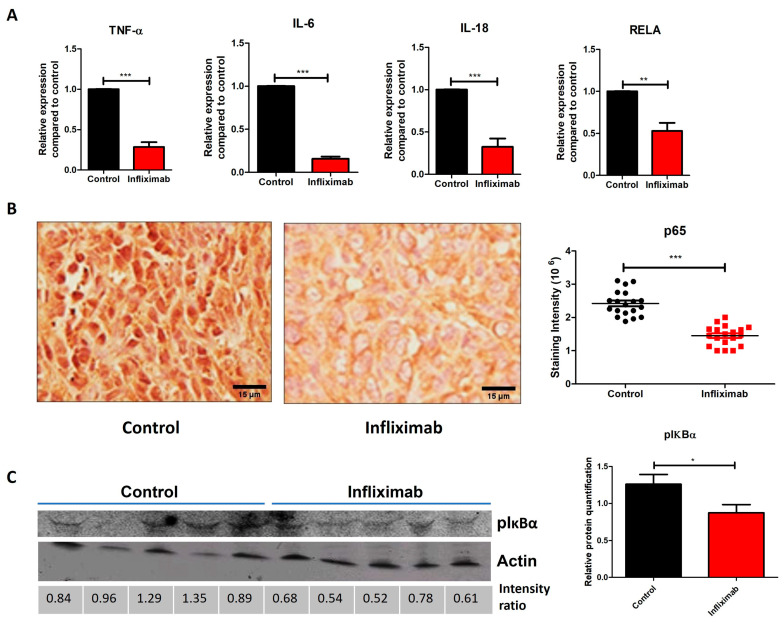
Infliximab inhibits NF-κB activation in breast tumors. (**A**) mRNA expression of *TNF-α*, *IL-6*, *IL-18* and *RELA* in Infliximab treated and control mice (n = 5). (**B**) Representative images of IHC staining using a specific antibody for RELA and nuclear staining by hematoxylin in Infliximab-treated mice compared to controls. (**C**) Immunoblot showing levels of pIκBα and actin using specific antibodies, determined by Western blotting, in the tumor sections of Infliximab-treated mice compared to controls. Uncropped blots are shown in Supplementary Figure S4. Significance is indicated by * *p* < 0.05, ** *p* < 0.01, and *** *p* < 0.001.

TNF-α binds to its receptor TNF-R1 and activates the NF-κB pathway, which regulates several key pathways essential for tumor progression. Therefore, we explored the effect of Infliximab on NF-κB activation and subsequent responses to inflammation. Inflammation-related genes such as those for *TNF-α*, *IL-6* and *IL-18* showed significant decreases in the mRNA levels in the primary tumors of Infliximab-treated mice compared to the controls. We also observed a significant decrease in levels of the *RELA* mRNA, the main subunit of the NF-κB dimer p65/p50 responsible for the activation of the pathway (Figure 3A). These RT-PCR results are compatible with those obtained by determining protein expression levels. There was a significant decrease in the mean intensity of immunohistology staining using an antibody against RELA (p65). Infliximab-treated mice also showed a decreased translocation of p65 from the cytoplasm to the nucleus (Figure 3B). The phosphorylation of IκBα by the IKK complex initiates its degradation and activates the nuclear translocation of p65, which is a major event in the activation of NF-κB. The level of the 36 kDa band of the phosphorylated form of IκBα decreased, which indicates decreased proteasomal degradation and decreased activation of NF-κB (Figure 3C). Taken together, these results from RT-PCR, IHC and Western blotting are consistent in demonstrating that Infliximab treatment inhibits NF-κB activation compared to controls.

### 3.4. TNF-α, RELA and ATX Are Positively Correlated in Breast Cancer Patients

**Figure 4:**
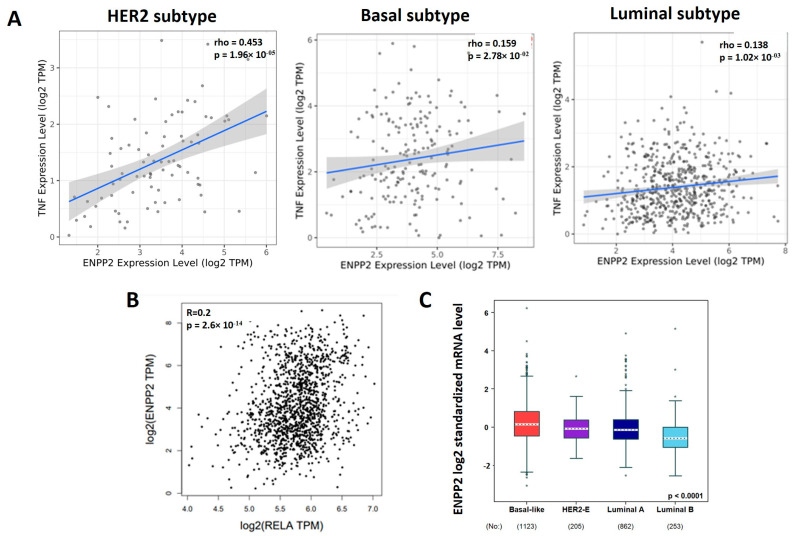
Positive correlations among *TNF-α*, *RELA* and *ATX* in breast cancer. (**A**) Positive correlation between *TNF-α* and *ATX* (*ENPP2*) in different subtypes of breast cancer patients. (**B**) Positive correlation between *RELA* and *ATX* in breast cancer patients. (**C**) Expression of *ENPP2* gene (*ATX*) in different subtypes of breast cancer patients.

We explored various databases to correlate the expression patterns of *TNF-α*, *NF-κB* signaling and ATX (*ENPP2*) expression in breast cancers. The Timer database, which is used for analyzing the correlation between genes in tumors, showed a positive correlation between *TNF-α* and *ENPP2* expression in different subtypes of breast cancer (Figure 4A). The HER2, Basal and Luminal A subtypes showed rho values equal to 0.453, 0.159 and 0.138, respectively. The Basal (highly metastatic) and Luminal A (less metastatic) subtypes both showed a positive correlation between *TNF-α* and *ENPP2* gene expression in patients. Moreover, there was a positive correlation between *RELA* (major NF-κB subunit) and *ENPP2* with an R value of 0.2 using the GEPIA2 database (TCGA and GTEX datasets) (Figure 4B). We also analyzed the bc-GenExMinor database for the expression of *ENPP2* in different subtypes of breast cancer patients (Figure 4C). *ENPP2* expression in tumors was significantly higher in the Basal (highly metastatic) subtype (n = 1123) in comparison to the HER2-E (n = 205), Luminal A (n = 862) and Luminal B (n = 253) subtypes. This suggests that the expression of *ATX* (*ENPP2*) is increased in a highly metastatic subtype of breast cancer. However, there was no significant association between patients that had high versus low *ENPP2* expression and survival rates using the GEPIA2 data base in the Basal subtype of breast cancer (Supplementary Figure S2). Thus, there were positive correlations among *TNF-α*, *RELA* and *ATX*, but these relationships need to be investigated further to understand their roles in the progression of breast cancer metastasis and patient survival.

### 3.5. TNF-α Induced NF-κB and ATX Activity Are Correlated in Breast Cancer

**Figure 5:**
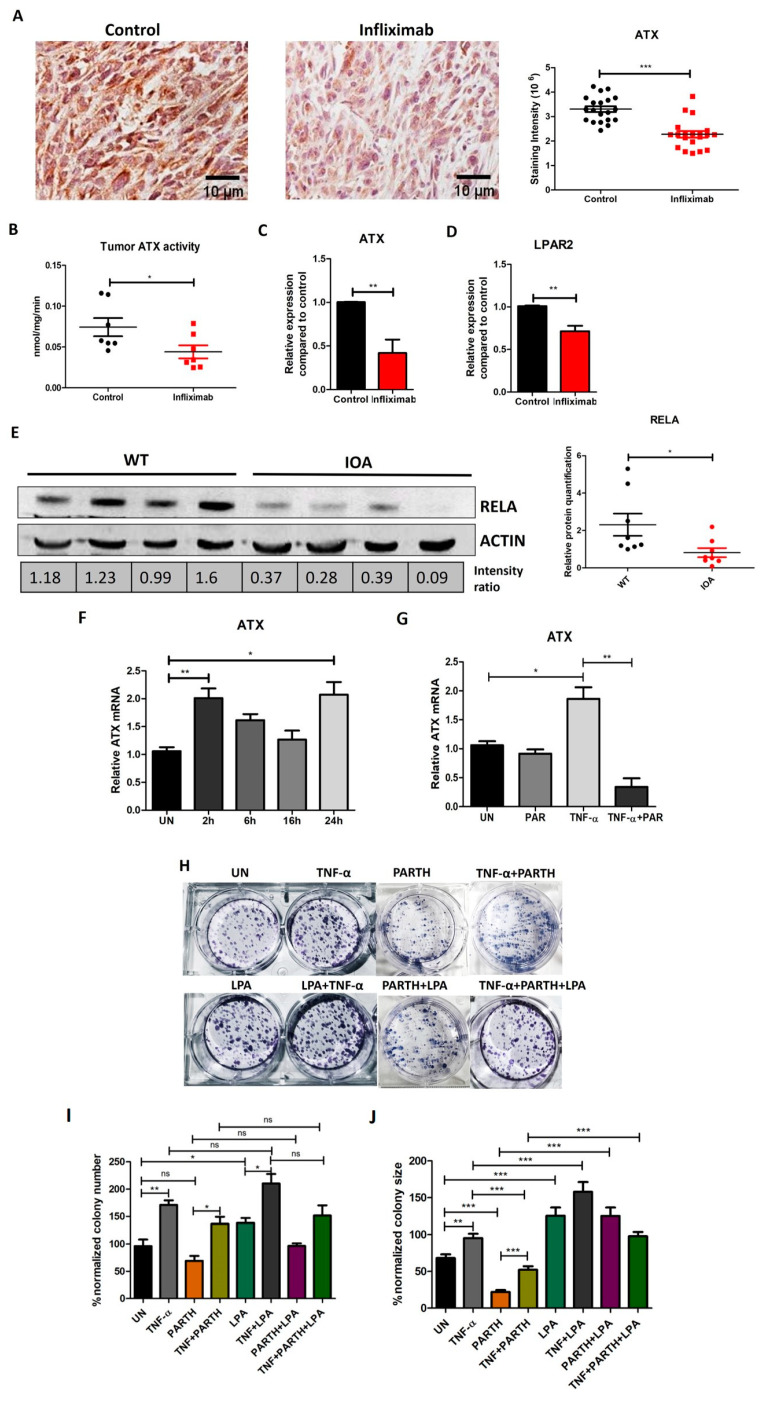
ATX and TNF-α-induced NF-κB activity are correlated in breast cancer and increased clone-forming ability. (**A**) IHC staining using an ATX-specific antibody in primary tumors from control and Infliximab-treated mice. (**B**) Tumor ATX activity in control and Infliximab-treated mice. (**C**,**D**) Comparison of ATX and LPAR2 mRNA expression in Infliximab and control mice via qRT-PCR analysis, respectively. (**E**) RELA levels in E0771 tumors of C57BL/c mice treated with the ATX inhibitor IOA-289 versus controls, determined by Western blotting. Uncropped blots are shown in Supplementary Figure S5. (**F**) Expression of ATX in 4T1 cells treated with TNF-α (10 ng/mL) for different time points, determined by qRT-PCR. (**G**) Expression of ATX in 4T1 cells treated with TNF-α (10 ng/mL) for 24 h and parthenolide (5 µM) for 4 h, determined by qRT-PCR. (**H**) Colony size of 4T1 cells treated with TNF-α (10 ng/mL), parthenolide (1 µM) and LPA (10 µM); measured using ImageJ. (**I**,**J**) Analysis of colony number and size for colony forming assay (n = 3), respectively. Significance is indicated by * *p* < 0.05, ** *p* < 0.01 and *** *p* < 0.001. ns = not significant.

The role of TNF-α in regulating ATX expression in breast cancer was further validated in the tumor tissues of Infliximab-treated mice. As established earlier, Infliximab decreases TNF-α-induced NF-κB activity, and so we evaluated TNF-α-induced ATX expression in these conditions. We observed a significant decrease in ATX staining via IHC using a specific antibody for ATX in the Infliximab-treated group (Figure 5A). In addition, ATX activity in the primary tumor tissue was also decreased in Infliximab-treated mice (Figure 5B). These changes are reflected in the Infliximab-induced decreases in mRNA for ATX (Figure 5C). LPAR2 receptor levels also showed decreases in the Infliximab group compared to the control group (Figure 5D). The LPAR1, LPAR3 and LPAR6 receptors and LPP1, LPP2 and LPP3 did not show significant changes (Supplementary Figure S3). Thus, these combined results show that blocking TNF-α activity in vivo decreased the expression of ATX in primary breast tumors.

We next determined the effects of blocking ATX activity in mice using the ATX inhibitor IOA-289, as described previously [30]. Using Western blotting, we observed a significant decrease in RELA expression in the tumors of IOA-289-treated mice compared to controls (Figure 5E). Furthermore, we investigated the role of TNF-α-induced NF-κB in regulating ATX expression by using 5 µM of parthenolide (an NF-κB inhibitor) in vitro. 4T1 cells showed upregulation in ATX expression upon treatment with TNF-α (10 ng/mL) for 24 h (Figure 5F). ATX expression decreased in 4T1 breast cancer cells in the presence of TNF-α and parthenolide for 4 h as compared to TNF-α alone (Figure 5G).

We further analyzed the clone-forming ability of 4T1 cells in different treatment conditions. TNF-α increased the number of clones formed by 4T1 cells compared to a control (Figure 5H,I). TNF-α, in combination with parthenolide, showed rescue when compared to parthenolide alone. The clone-forming ability was not affected significantly by LPA alone, but there was an increase in combination with TNF-α. This appeared to be decreased by parthenolide, but it did not reach the level of significance (*p* value = 0.084). To analyze these results further, we also measured the average colony sizes and observed an increase in the presence of TNF-α, LPA or their combination compared to the untreated control (Figure 5J). The average colony size decreased in the presence of 1 µM of parthenolide and was partially rescued by the addition of 10 ng/mL of TNF-α and 10 µM of LPA or the combination of treatments given until the termination of incubation, as described in the Section 2. These results strengthen our hypothesis that the activation of NF-κB can increase the expression of ATX in breast cancer and thereby increase proliferation and metastatic potential. Together, these results show that TNF-α-induced NF-κB activation increases limited ATX expression in breast cancer cells, and LPA production increases clonogenicity.

## 4. Discussion

Inflammation-inducing factors in the TME can cause adverse effects on the metastasis and treatment of breast cancers [39,40]. TNF-α has a dual role in breast cancer, and its effects are mainly regulated depending on concentrations in the tumor and the TME, and this is responsible for breast cancer cell survival or cell death [40]. TNF-α can be produced by both the breast cancer cells and immune cells in the TME, which regulates diverse processes such as cell–cell communication, proliferation, differentiation and metastasis and cell death [41]. TNF-α-induced inflammation is associated with disease severity and a higher rate of recurrence in breast cancer [14]. Biopsy samples of breast tumors show increased TNF-α expression, and this has been associated with a worse prognosis [42]. Despite intensive research into TNF-α-induced effects in breast cancer biology, details of the molecular mechanisms of TNF-α action remain largely unexplored. In the current study, we report that the inhibition of TNF-α signaling in a syngeneic mouse model of breast cancer using Infliximab inhibited metastasis to the lungs by inhibiting NF-κB and LPA-ATX signaling.

We used Infliximab, a chimeric human–mouse monoclonal antibody which consists of human IgG1 Fc region fused with murine Fv region, against TNF-α. The antibody binds specifically to TNF-α and prevents interaction with TNF-α receptors. Earlier, Infliximab-based studies showed a remarkable decrease in the metastasis of osteosarcoma cells to lungs [32]. Related studies in advanced cancer showed that Infliximab was well tolerated with few toxic effects, and it decreased inflammatory factors including CCL2, IL-6 and serum CRP [43]. Our results established that Infliximab-treated mice showed marginally reduced breast tumor weight compared to control mice. Our major finding was a ~60% reduction in breast cancer metastasis to lungs in an Infliximab-treated group. Moreover, a significant decrease was observed in metastatic and proliferation markers such as tumor Vimentin and Ki-67, respectively, in the Infliximab-treated group. High Ki-67 expression has been well corelated with breast cancer subtypes and relapse in patients [44]. Studies related to Vimentin in breast cancer cells have considered it a new target for therapeutics and have shown it to be associated with the recurrence of triple-negative breast cancer (TNBC) [45]. Overall, these findings, together with our present study, validate that blocking TNF-α signaling can decrease breast tumor growth and have an even more marked effect in decreasing metastasis.

The correlation among inflammation, cytokine and chemokines produced in the tumor to promote growth and metastasis has been studied extensively [8,46]. Crosstalk among different cell types in the TME shows that cytokines like IL-1β, produced by activated fibroblasts, create a tumor-promoting environment for ER-positive breast cancer cell growth [47]. IL-6 expression in 249 patient serum samples was significantly increased, and this was associated with the recurrence risk of breast cancer [48]. TNF-α-induced NF-κB activation regulates the expression of multiple inflammation factors, which leads to a cytokine milieu in the TME that is required for tumor progression. A marked increase occurs in the expression of inflammatory cytokines such as IL-6, IL-8 and TGF-β upon TNF-α stimulation in TNBC. Such events also trigger the expression of EMT markers and increase metastatic potentials [49]. We investigated the effects of Infliximab on the concentrations of 32 cytokines, chemokines and growth factors in plasma and primary tumors. We observed decreases in the TNF-α, IL-4 and MCP-1 and an increase in IFNγ levels in the plasma of the Infliximab group compared to controls. Patient studies involving 768 individuals with varying genotypes have shown that TNF-α is a critical player in tumor progression and distant metastasis, especially in TNBC [50]. Decreased serum levels of TNF-α were also associated with anti-tumor effects in an MCF-7-derived breast cancer in a mouse model treated with the opiate, tramadol, which is associated with reduced mortality in patients [51]. TNF-α gene knockout studies in TNBC cell lines show inhibited cell proliferation and induced apoptosis [52]. IL-4 signaling was identified to trigger tumor-associated macrophages and enhance invasion in breast cancer [53]. Dutta et al. showed that MCP-1 is increased in TNBC cell lines compared to ER-positive MCF-7 cells and explored its role in cell invasion and increasing EMT markers like N-cadherin and Vimentin [54]. IFN-γ, which was increased by Infliximab in the serum in our experiments, was shown to have anti-tumor effects by decreasing cancer stem cell numbers in a 4T1 mouse model of breast cancer [55].

Furthermore, IL-4 and IL-12p40 were also decreased in the breast tumors by Infliximab treatment. Human breast cancer cells produce IL-12p40 as a mechanism to suppress anti-tumor immune responses and protect the tumor from the immune system [56]. Other studies showed that decreasing inflammatory cytokines has a promising role in controlling breast cancer invasion and metastasis [57]. This profiling identified potential candidates that can contribute to the invasiveness and metastatic potentials of breast cancer cells, and these are mainly regulated by the NF-κB pathway.

Next, we experimentally verified the activation of the NF-κB pathway in the primary breast tumors, which is the major pathway activated upon TNF-α stimulation. NF-κB activation is a typical phenomenon detected in breast cancer [58]. The genes that are altered by NF-κB activation are often prime sources of disease aggressiveness and poor prognosis [59,60]. We analyzed inflammation-related genes, such as those for TNF-α, IL-6, IL-18 and RELA, which showed reduced mRNA levels in the tumors of Infliximab-treated mice. IL-6 has been identified to promote tumorigenesis and metastasis in breast cancer. It is considered as a key cytokine effecting STAT3 phosphorylation in many cancers, and it is associated with a poor prognosis [61,62]. Engineered bispecific antibodies that target IL-6 decrease the migratory potential of metastatic TNBC [63].

IL-18 expression was decreased after Infliximab treatment. IL-18 is essentially a pro-inflammatory cytokine that activates NF-κB [64], and it has a dual effect in tumor progression: it can activate immune responses and eliminate cancer cells, whereas higher levels of IL-18 promote angiogenesis, metastasis, proliferation and immune escape [65]. Serum IL-18 levels in patients with gastric cancer and skin cutaneous melanoma have been correlated with cell migration and malignancy [66,67]. High levels of IL-18 are also found in breast cancer patients with metastasis [68,69]. IL-18 plays an important role in inducing breast cancer cell migration by activating the p38 MAPK pathway and downregulating claudin-12 [70].

We also validated the involvement of NF-κB activation using the nuclear translocation of p65. RELA translocates to the nucleus to activate NF-κB signaling. Kanzaki et al. showed that inhibiting the nuclear translocation of RELA decreases the growth of TNBC [71]. The ubiquitination of p65 (RELA) and its degradation by E3 ligase, FBXW2, leads to suppressed breast cancer stemness and tumorigenesis [72]. IHC staining revealed a reduced colocalization of p65 with the nucleus in Infliximab-treated mice. Furthermore, reduced TNF-α in the plasma of the Infliximab-treated mice was associated with a decreased phosphorylated of IκBα which, upon ubiquitination and proteosomal degradation, activates the NF-κB pathway. These results establish that NF-κB activation was decreased in the Infliximab-treated mice compared to the controls.

The cross-talk among different pathways in breast cancer and their cumulative effects on cell death or survival needs extensive investigation. Therefore, we focused on the ATX-LPA-inflammatory cycle, which drives breast tumor growth and metastasis. ATX expression is stimulated by inflammatory cytokines [23]. ATX secretion is stimulated after tissue injury and promotes wound healing by increasing cell migration and division. ATX achieves this by producing lysophosphatidate (LPA), which signals through six G-protein-coupled receptors. Inflammation during wound healing increases the secretion of ATX and consequent LPA production, which then activates NF-κB and stimulates the production of more inflammatory cytokines in a feedforward cycle [73]. This inflammatory cycle is resolved under normal circumstances, but chronic activation of the ATX-LPA-inflammatory axis in cancers leads to enhanced tumor growth and especially metastasis [74]. Increased LPA signaling has been associated with metastatic tumors and breast tumorigenesis [75]. Previous studies from our group emphasized the importance of ATX in the TME and established that inhibiting ATX can reduce breast cancer metastasis to lungs [76]. We investigated whether the TNF-α-regulated NF-κB pathway can control the expression of ATX, which is one of the upregulated genes in breast cancer [24]. Basal and TNF-α-induced NF-κB activation and ATX expression also play important roles in liver tumorigenesis [29]. Thus, it was important to dissect the role of TNF-α-induced inflammation in ATX-LPA signaling in breast cancer.

First, we explored cancer databases and established that TNF-α, RELA and ATX in breast tumors showed positive correlations in patients. Increased expression of the ENPP2 (ATX) gene was observed in highly metastatic TNBC patients compared to less metastatic ones. However, there was no significant relationship between disease-free survival or the overall survival of patients with high versus low levels of tumor ATX (ENPP2) expression in the GEPIA2 database. Other studies, which involved our group, established that ATX in human breast tumors is produced mainly in the TME by fibroblasts and endothelial cells rather than by breast cancer cells [77]. Significantly, stromal composition correlates poorly with the subtype or grade of tumors [78]. Since the majority of tumors in the TIMER data base that was used in this study were early and/or non-metastatic, such tumors can be regarded as “early wounds” within the surrounding breast parenchyma [79]. ATX is a major factor that promotes wound healing [74], and in early breast cancer, it appears to mitigate tumor growth [77]. Conversely, in later stages of breast cancer with high ATX expression, there is an increased expression of gene sets that drive tumor stemness, survival against treatment and progression to metastasis [77]. Thus, the actions of ATX are complicated: ATX can promote wound healing in early stages of breast cancer but then becomes maladaptive to promote treatment resistance, immune evasion and metastasis as the cancer progresses [26,74].

These observations strengthened our hypothesis that maladaptive ATX-LPA signaling is driven by an inflammatory cycle, implying that decreasing inflammation should attenuate ATX-LPA signaling. We already showed this effect by treating mice with dexamethasone and establishing that this decreased ATX-dependent LPA signaling [80] and the effects of radiation in activating this pathway [81]. Dexamethasone is a very effective anti-inflammatory agent, but it has limited application in cancer therapy because of its side effects and immune-suppressive properties. Therefore, our choice of Infliximab for our present studies is much more relevant to cancer therapy.

As expected, we observed a decrease in ATX staining in tumors from Infliximab-treated mice together with decreased ATX activity and mRNA levels. Significantly, Infliximab treatment also decreased the mRNA for LPAR2 in breast tumors. These changes were accompanied by decreased lung metastasis. Previous studies also showed that downregulating endogenous ATX in 4T1 cells reduces osteolytic bone metastasis in BALB/c mice [75].

To further establish a link between ATX and NF-κB, we measured the expression of RELA after ATX inhibition in mice with IOA-289, which is now in a Phase 1B trial for treating pancreatic cancer. Interestingly, IOA-289 decreased RELA expression in breast tumors. This observation establishes an important link between the TNF-induced NF-κB pathway and ATX expression in breast cancer. It also provides a further explanation for why IOA-289, an ATX inhibitor, decreases the growth of breast tumors and metastasis in two mouse models [30,76]. These actions of IOA-289 were linked to increased numbers of CD8-T-cells in the tumors.

We also established that TNF-α increases ATX expression in the 4T1 breast cancer cell line and increases the clone-forming ability, which depends on the activation of NF-κB. It should be noted that ATX expression is low in breast cancer cells and that other tumor cells, including fibroblasts, leukocytes and endothelial cells, are probably much more important in the ATX production that drives breast tumor growth and metastasis in mice [80]. The activation of NF-κB upon TNF-α treatment is a time-dependent mechanism. The NF-κB pathway often shows a response that is divided into an early phase, a middle phase and a late phase. Many of the NF-κB genes show biphasic responses [82,83], and this probably explains the changes in ATX expression over time after TNF-α treatment. Also, TNF-α and LPA, in the present work, increased the size of clones formed by 4T1 breast cancer cells, which are highly metastatic.

Taken together, this study highlights the role of TNF-α-induced NF-κB activation in regulating inflammatory signaling and explores new molecular insights that can attenuate the invasiveness and metastatic properties of breast tumors. In particular, we show that the effects of TNF-α-induced inflammation are mediated partly by increased ATX expression and LPA signaling, which increases the metastatic potential of tumors. This work provides an increased understanding of how to regulate the ATX-LPA-inflammatory cycle and prevent it from becoming maladaptive and pro-metastatic. This concept could be developed as a novel treatment for improving the outcomes for breast cancer patients by using infliximab to decrease the effects of TNF-α together with ATX-LPA inhibitors.

## 5. Conclusions

Our work concentrated on the role of the feed-forward cycle whereby inflammation increases the secretion of ATX, which produces LPA that then induces more inflammation. This cycle is pro-metastatic. It was studied in an orthotopic syngeneic model of breast cancer in which inflammation was decreased by blocking the actions of TNFα with Infliximab. This treatment decreased the activation of the NF-κB pathway and the expression of inflammatory-related genes including ATX, which resulted in a decrease of ~60% in lung metastases. This major finding identifies TNF-α as a critical component of inflammatory-induced metastasis. TNF-α, NF-κB signaling and ATX expression are positively linked to each other in breast cancer patients, and this pathway plays a role in increasing the clonogenicity of breast cancer cells. Overall, the present study shows that the effects of the ATX-LPA-inflammatory cycle on metastasis can be attenuated by blocking TNF-α signaling. This strategy could be used in future studies in combination with existing chemotherapeutic agents, radiotherapy or novel ATX inhibitors to study their efficacy in decreasing cancer metastasis.

## Data Availability

Data are contained within the article and Supplementary Materials.

## Supplementary Materials

The following are available online at https://www.mdpi.com/article/10.3390/cancers16010052/s1: Figure S1. Validation of mRNA measurements for *IL-6* in tumors using *cyclophilin A*, *GAPDH* and *β-actin* as housekeeping genes for Infliximab-treated and control mice; Figure S2: Kaplan–Meier survival curves of disease-free and overall survival for ENPP2 (ATX) in breast cancer patients; Figure S3: mRNA expression for *LPAR* and *LPPs*; Figure S4: Uncropped blots for Figure 3C; pIκBα and Actin; Figure S5: Uncropped blots for Figure 5E; RELA and Actin; Table S1: Primer Details; Table S2: Datasets used for the GenEx Miner database.

## Abbreviations

ATX	Autotaxin
EMT	Epithelial–mesenchymal transition
IL	Interleukin
LPA	Lysophosphatidic acid, lysophosphatidate
LPAR	LPA receptor
LPP	Lipid phosphate phosphatase
NF-κB	Nuclear factor kappa-light-chain-enhancer of activated B cells
TNF-α	Tumor Necrosis Factor alpha
RELA	RELA protooncogene (NF-κBsubunit)
TME	Tumor microenvironment
