# Supplementary material for: Infliximab, a Monoclonal Antibody against TNF-α, Inhibits NF-κB Activation, Autotaxin Expression and Breast Cancer Metastasis to Lungs
Source: Cancers (Basel). 2023 Dec 21;16(1):52. doi: 10.3390/cancers16010052 (PMC10778319; doi:10.3390/cancers16010052)
Supplement: Supplementary file 1 [file cancers-16-00052-s001.zip › Supplementary Figure S1.pdf]

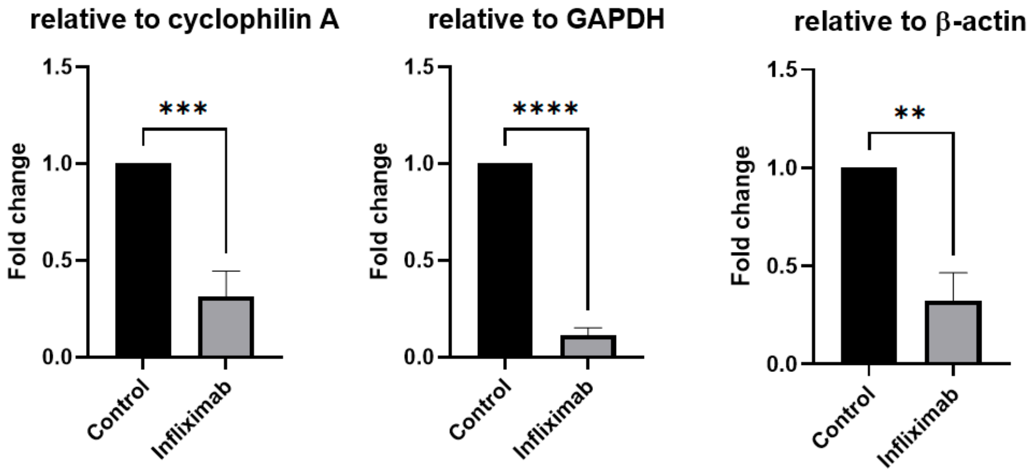

Supplementary Figure S1. Validation of mRNA measurements for *IL-6* in tumors using *cyclophilin A, GAPDH* and β-actin as housekeeping genes for Infliximab treated and control mice (n=5). Results using the three housekeeping genes provide essentially the same conclusions.
