# Supplementary material for: Infliximab, a Monoclonal Antibody against TNF-α, Inhibits NF-κB Activation, Autotaxin Expression and Breast Cancer Metastasis to Lungs
Source: Cancers (Basel). 2023 Dec 21;16(1):52. doi: 10.3390/cancers16010052 (PMC10778319; doi:10.3390/cancers16010052)
Supplement: Supplementary file 1 [file cancers-16-00052-s001.zip › Supplementary Figure S2.pdf]

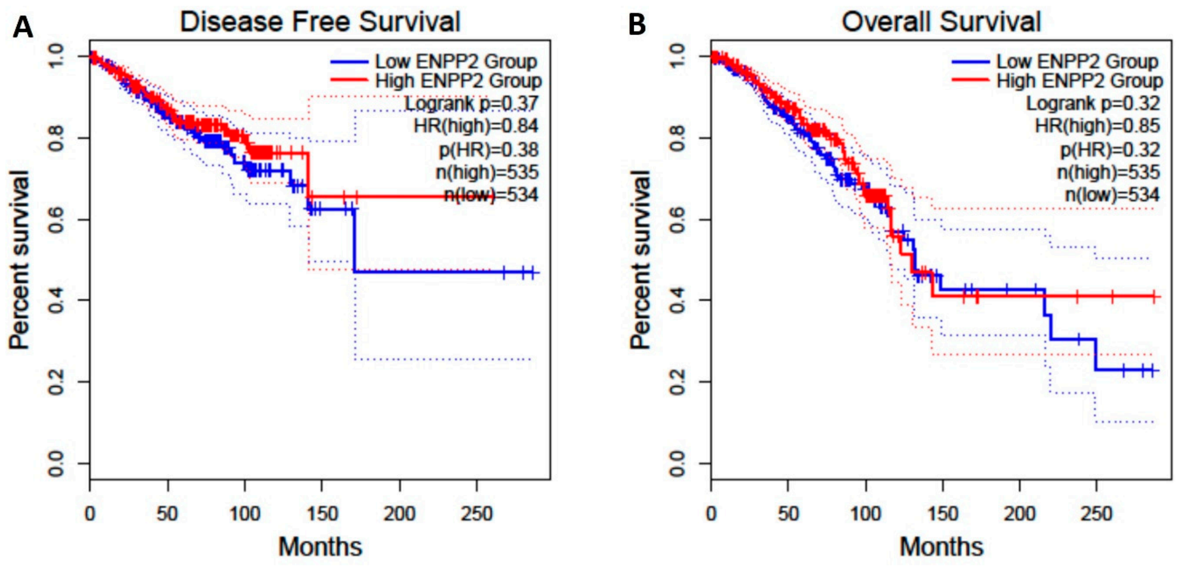

Supplementary Figure S2. Kaplan-Meier survival curves of disease free and overall survival for ENPP2 (ATX) in breast cancer patients. Survival curves are represented as solid lines, and the dotted lines represents the 95% confidence interval. The GEPIA2 database was used for the analysis.
