# Supplementary material for: Infliximab, a Monoclonal Antibody against TNF-α, Inhibits NF-κB Activation, Autotaxin Expression and Breast Cancer Metastasis to Lungs
Source: Cancers (Basel). 2023 Dec 21;16(1):52. doi: 10.3390/cancers16010052 (PMC10778319; doi:10.3390/cancers16010052)
Supplement: Supplementary file 1 [file cancers-16-00052-s001.zip › Supplementary Figure S3.pdf]

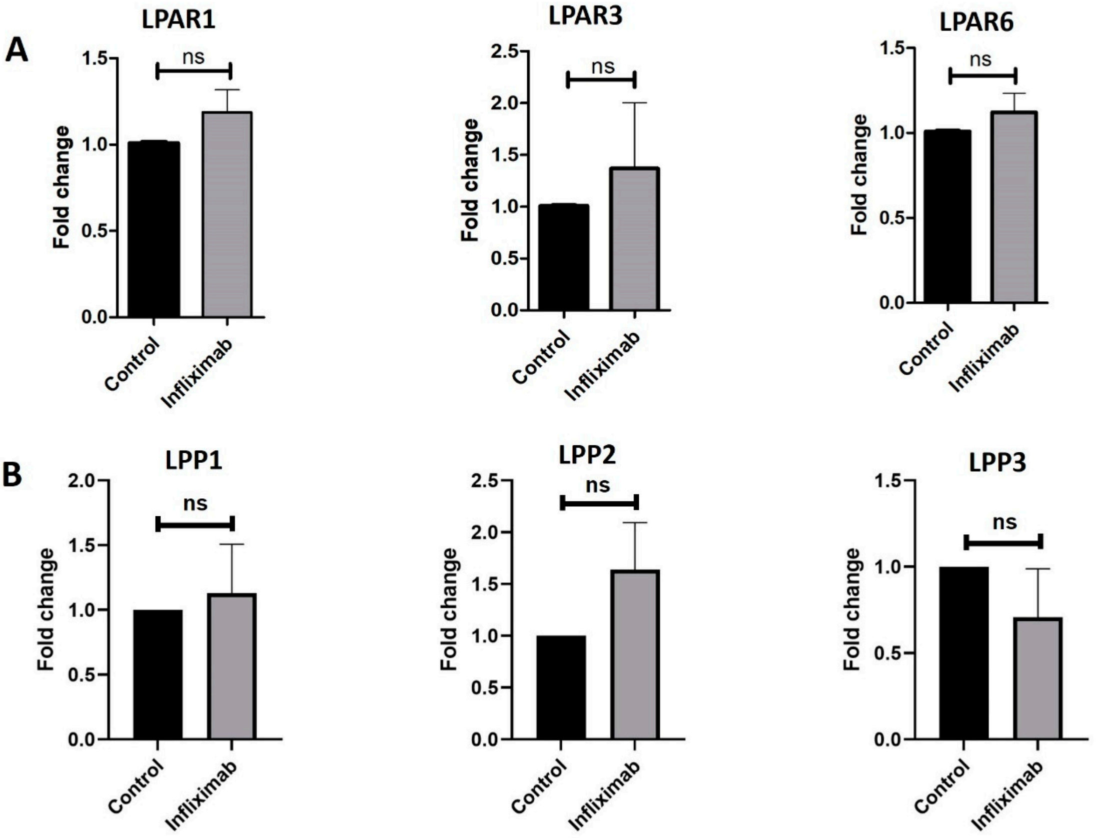

Supplementary Figure S3. mRNA expression for *LPAR* and *LPPs*. (**A**&**B**) mRNA expression for LPAR1, LPAR3, LPAR6 receptor levels and LPP1, LPP2, LPP3 in Infliximab group compared to control respectively (n=5). Similar results were calculated for LPP1, LPP2 and LPP3 when we used glyceraldehyde phosphate dehydrogenase or β-actin as reference genes.
