# Supplementary material for: Infliximab, a Monoclonal Antibody against TNF-α, Inhibits NF-κB Activation, Autotaxin Expression and Breast Cancer Metastasis to Lungs
Source: Cancers (Basel). 2023 Dec 21;16(1):52. doi: 10.3390/cancers16010052 (PMC10778319; doi:10.3390/cancers16010052)
Supplement: Supplementary file 1 [file cancers-16-00052-s001.zip › Supplementary Table S1- Primer details.pdf]

Supplementary Table S1. Primer Details:

Mouse RELA Forward	ACCCGAAACTCAACTTCTGTC
Mouse RELA Reverse	TTGATGGTGCTGAGGGATG
Mouse TNF-α Forward	CTTCTGTCTACTGAACTTCGGG
Mouse TNF-α Reverse	CAGGCTTGTCACTCGAATTTTG
Mouse IL-6 Forward	CAAAGCCAGAGTCCTTCAGAG
Mouse IL-6 Reverse	GTCCTTAGCCACTCCTTCTG
Mouse IL-18 Forward	GCCTCAAACCTTCCAAATCAC
Mouse IL-18 Reverse	GTTGTCTGATTCCAGGTCTCC
Mouse ENPP2 (ATX) Forward	GAAAGCAGAGCATTCAGGGC
Mouse ENPP2 (ATX) Reverse	GGATATTACCTGGTATGACCCGAAA
Mouse LPAR1 Forward	CTATGTTCGCCAGAGGACTATG
Mouse LPAR1 Reverse	GCAATAACAAGACCAATCCCG
Mouse LPAR2 Forward	CACACTCAGCCTAGTCAAGAC
Mouse LPAR2 Reverse	GTACTTCTCCAGAGCCAGAAC
Mouse LPAR3 Forward	GCCCGGTGTGCAATAAAA
Mouse LPAR3 Reverse	CTTAAAAGCCCCAGAAGTGATG
Mouse LPAR6 Forward	CACATCTGAATAGCAAAGGCG
Mouse LPAR6 Reverse	TGAACATGCACCCGTACAG
PLPP1 (LPP1) H/M Forward	GGTCAAAAATCAACTGCAG
PLPP1 (LPP1) H/M Reverse	TGGCTTGAAGATAAAGTGC
PLPP2 (LPP2) H/M Forward	TGGCCAAGTACATGATTGG
PLPP2 (LPP2) H/M Reverse	AGCAGCCGTGCCCACTTCC
PLPP3 (LPP3) H/M Forward	CCCGGCGCTCAACAACAACC
PLPP3 (LPP3) H/M Reverse	TCTCGATGATGAGGAAGGG
Mouse cyclophilin A Forward	CACCGTGTTCTTCGACATCAC
Mouse cyclophilin A Reverse	CCAGTGCTCAGAGCTCGAAAG
Mouse GAPDH Forward	ACTTTGTCAAGCTCATTTCC
Mouse GAPDH Reverse	TCTTACTCCTTGGAGGCCAT
Mouse β-actin Forward	ACCTTCTACAATGAGCTGCG
Mouse β-actin Reverse	CTGGATGGCTACGTACATGG
