# Supplementary material for: Infliximab, a Monoclonal Antibody against TNF-α, Inhibits NF-κB Activation, Autotaxin Expression and Breast Cancer Metastasis to Lungs
Source: Cancers (Basel). 2023 Dec 21;16(1):52. doi: 10.3390/cancers16010052 (PMC10778319; doi:10.3390/cancers16010052)
Supplement: Supplementary file 1 [file cancers-16-00052-s001.zip › Supplementary Table S2- bc-GenExMiner datasets.pdf]

**Supplementary Table S2: bc-GenExMiner datasets used:**						
Ver	Study code	Original data		Filtered data		Final data
		Reference	No. patients	Intrinsic molecular subtypes -selected patients	Gene found	No. patients
1	Rosetta2002	Van de Vijver et al., 2002	295	295		295
1	PNAS1732912100	Sotiriou et al., 2003	99	99		98
1	GSE1379	Ma et al., 2004	59	59		59
1	GSE2603	Minn et al., 2005	82	82		82
1	GSE1456	Pawitan et al., 2005	159	159		159
1	GSE2034	Wang et al., 2005	286	286		286
1	GSE2741	Weigelt et al., 2005	50	50		49
1	GSE3143	Bild et al., 2006	158	158		158
1	E_TABM_158	Chin et al., 2006	112	112		112
1	GSE4922	Ivshina et al., 2006	249	249		249
1	GSE7390	Desmedt et al., 2007	198	198		198
1	GSE6532	Loi et al., 2007	267	267		267
1	GSE5327	Minn et al., 2007	58	58		58
1	E_UCON_1	Naderi et al., 2007	135	135		135
1	GSE7849	Anders et al., 2008	75	75		75
1	GSE9893	Chanrion et al., 2008	151	151		151
1	GSE9195	Loi et al., 2008	77	77		77
1	GSE10510	Calabrò et al., 2009	139	139		139
1	GSE11264	Jézéquel et al., 2009	252	0		0
1.1	GSE11121	Schmidt et al., 2008	200	200		200
1.1	GSE12093	Zhang et al., 2009	136	136		136
3.1	GSE8757	Chin et al., 2007	171	171		171
3.1	GSE7378	Zhou et al., 2007	54	54		54

3.1	GSE16391	Desmedt et al., 2009	55	55		55
3.1	GSE22133	Jönsson et al., 2010	346	345		345
3.1	GSE19615	Li et al., 2010	115	115		115
3.1	GSE17907	Sircoulomb et al., 2010	55	55		55
3.1	GSE22219	Buffa et al., 2011	216	216		216
3.1	GSE20711	Dedeurwaerder e t al., 2011	85	85		85
3.1	GSE26971	Filipits et al., 2011	277	277		277
3.1	GSE25055	Hatzis et al., 2011	309	309		309
3.1	GSE20685	Kao et al., 2011	296	296		296
3.1	GSE21653	Sabatier et al., 2011	239	239		239
3.1	GSE16987	Wang et al., 2011	149	149		149
3.1	GSE45255	Nagalla et al., 2013	41	41		41
4.3	GSE2109	expO et al., 2005	298	298		298
4.3	GSE8193	Yau et al., 2007	47	47		47
4.3	GSE20462	Parris et al., 2010	94	94		94
4.3	GSE17705	Symmans et al., 2010	43	43		43
4.3	GSE24450	Heikkinen et al., 2011	174	174		174
4.3	GSE31448	Sabatier et al., 2011	71	71		71
4.3	METABRIC	Curtis et al., 2012	1 980	1 980		1 980
4.3	E_MTAB_365	Guedj et al., 2012	536	536		536
4.3	GSE30682	Servant et al., 2012	343	343		343
4.3	GSE42568	Clarke et al., 2013	104	104		104
4.3	GSE40115	Larsen et al., 2013	183	183		183
4.3	GSE55348	Castagnoli et al., 2014	53	53		53
4.3	GSE43358	Fumagalli et al., 2014	56	56		56
4.3	GSE36295	Merdad et al., 2014	45	45		45
4.3	GSE37751	Terunuma et al., 2014	55	55		55
4.3	GSE76274	Burstein et al., 2015	66	66		66
4.3	GSE97177	Biermann et al., 2017	53	53		53

4.5	GSE12276	Bos et al., 2009	204	204		204
4.5	GSE18864	Silver et al., 2010	75	75		75
4.6	GSE80999	Aure et al., 2017	381	381		381
4.6	GSE86166	Prabhakaran et al., 2017	366	366		366
Total:			10 872	10 619	55	10 617
