# Supplementary figures and images for: Infliximab, a Monoclonal Antibody against TNF-α, Inhibits NF-κB Activation, Autotaxin Expression and Breast Cancer Metastasis to Lungs
Source: Cancers (Basel). 2023 Dec 21;16(1):52. doi: 10.3390/cancers16010052 (PMC10778319; doi:10.3390/cancers16010052)

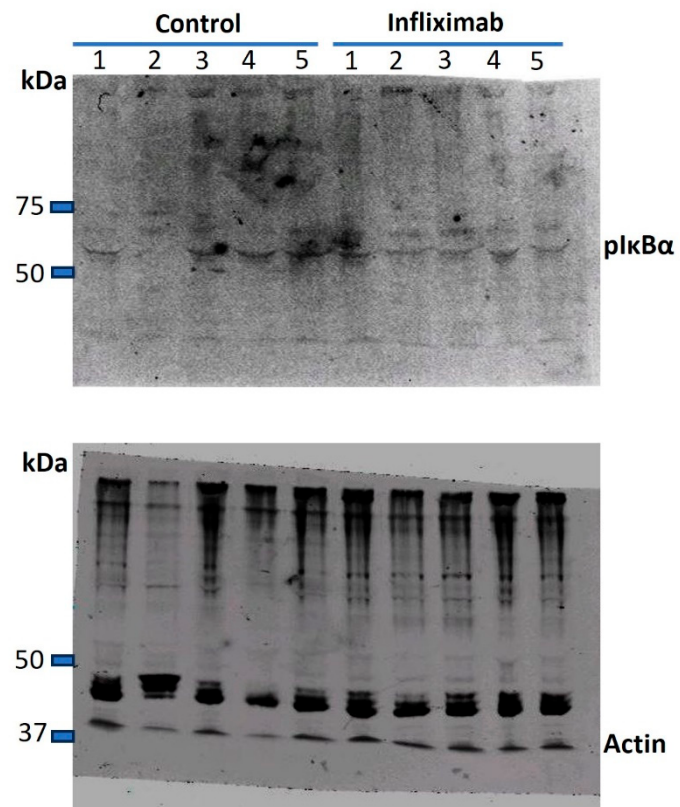

**Supplementary Figure S4.** Uncropped blots for Figure 3C; pIκBα and Actin.

Supplement: Supplementary file 1 [file cancers-16-00052-s001.zip › Supplementary Figure S4.pdf]

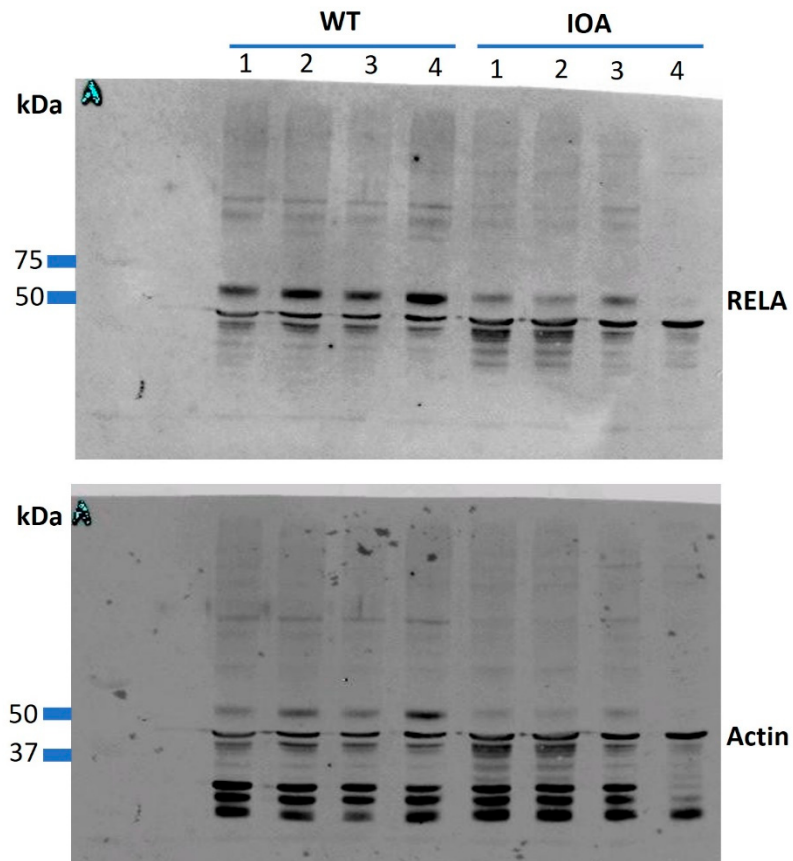

**Supplementary Figure S5.** Uncropped blots for Figure 5E; RELA and Actin.

Supplement: Supplementary file 1 [file cancers-16-00052-s001.zip › Supplementary Figure S5.pdf]
